# Health Risk Assessment of Toluene and Formaldehyde Based on a Short-Term Exposure Scenario: A Comparison of the Reference Concentration, Reference Dose, and Minimal Risk Level

**DOI:** 10.3390/toxics13080683

**Published:** 2025-08-16

**Authors:** Ji-Eun Moon, Si-Hyun Park, Young-Hyun Kim, Hyeok Jang, Ji-Yun Jung, Sung-Won Yoon, Cheol-Min Lee

**Affiliations:** Department of Environmental and Chemical Engineering, Seokyeong University, 124, Seogyeong-ro, Seongbuk-ku, Seoul 02713, Republic of Korea; mje0313@skuniv.ac.kr (J.-E.M.); shp8880@skuniv.ac.kr (S.-H.P.); rladudgus128@skuniv.ac.kr (Y.-H.K.); amer1can@skuniv.ac.kr (H.J.); jju1049@skuniv.ac.kr (J.-Y.J.); ss5372@skuniv.ac.kr (S.-W.Y.)

**Keywords:** chemical accident, health risk assessment, minimal risk level, reference concentration, reference dose

## Abstract

Conventional health risk assessments do not adequately reflect short-term exposure characteristics following chemical accidents. We aimed to evaluate the efficacy of existing assessment methods and propose a more suitable risk assessment approach for short-term exposure to hazardous chemicals. We analyzed foundational studies used to derive reference concentration (RfC), reference dose (RfD), and minimal risk level (MRL) values and applied these health guidance values (HGVs) to a hypothetical chemical accident scenario. An analysis of the studies underlying each HGV revealed that, except for the RfC for formaldehyde and the RfD for toluene, all values were derived under research conditions comparable to their respective exposure durations. Given the differing toxicity mechanisms between acute and chronic exposures, MRLs that were aligned with the corresponding exposure durations supported more appropriate risk management decisions. The health risk assessment results showed that RfC/RfD-based hazard quotients (HQs) were consistently higher than MRL-based HQs across all age groups and both substances, indicating that RfC/RfD values tend to yield more conservative risk estimates. For formaldehyde, the use of RfC instead of MRL resulted in an additional 208 tiles (2.08 km^2^) being classified as areas of potential concern (HQ > 1) relative to the MRL-based evaluation. These findings highlighted that the selection of HGVs can significantly influence the spatial extent of areas of potential concern, potentially altering health risk determinations for large population groups. This study provides a scientific basis for improving exposure and risk assessment frameworks under short-term exposure conditions. It also serves as valuable foundational data for developing effective and rational risk management strategies during actual chemical accidents. To the best of our knowledge, this is the first study to apply MRLs to a short-term chemical accident scenario and directly compare them with traditional reference values.

## 1. Introduction

With growing economic development and industrialization in modern society, the volume of chemical substances required for certain industrial processes has significantly increased, leading to the continued occurrence of chemical accidents [1]. These incidents cause localized damage at the accident site and often affect the surrounding environment [2], causing extensive human and material losses, environmental contamination, and harm to local communities [3]. Consequently, considerable efforts have been made to develop tools to assess the health risks associated with exposure to hazardous chemicals released during accidents. For example, Park et al. [4] proposed a health risk assessment methodology to evaluate the potential long-term health effects of hazardous chemical exposure among residents living near chemical accident sites, aiming to support both damage prevention and post-accident management. This methodology adheres to the four-step health risk assessment framework devised by the United States National Research Council and the United States National Academy of Sciences, as adopted by the United States Environmental Protection Agency (US EPA) [5]. To reflect the fate and transport characteristics of hazardous chemicals released during an accident, the exposure assessment component incorporates multimedia transport modeling, accounting for intermedia transfer and degradation half-lives to estimate the time until dissipation in each medium. The total exposure dose is then calculated for the period between the time of release and dissipation. The health risk is subsequently assessed using health guidance values (HGVs), which are defined as exposure levels deemed sufficiently protective of human health for a given duration and exposure route; specifically, the reference concentration (RfC) and reference dose (RfD) values determined by the US EPA. However, RfC and RfD are estimates of exposure levels at which no adverse effects are expected over a lifetime of continuous exposure [6,7]. As such, applying these chronic values to hazardous substances with short-term exposure characteristics, such as those involved in chemical accidents, may introduce limitations in accurately characterizing health risks.

Although the US EPA is developing the acute reference exposure (ARE) methodology to establish HGVs for exposures ≤24 h, no guidance currently exists for exposures lasting >1 d, i.e., several days [7]. In contrast, the Agency for Toxic Substances and Disease Registry (ATSDR) has developed minimal risk levels (MRLs), defined as daily human exposure estimates below which adverse non-cancer health effects are not expected to occur. MRLs are categorized by exposure duration (acute [1–14 d], intermediate [15–364 d], and chronic [≥1 year]), and are provided for both inhalation and oral exposure routes [8]. Despite the availability of MRLs, most health risk assessments in practice continue to rely on RfC/RfD values, with limited application of MRLs. Chronic values such as RfC and RfD are often applied to short-term exposure scenarios, creating a potential mismatch between actual exposure characteristics and the applied HGVs. This mismatch may result in either underestimating or overestimating health risks, underscoring the need for a systematic comparison between the two HGV types to evaluate how their application may influence risk outcomes.

Therefore, this study aimed to analyze the foundational studies used to derive RfC/RfD and MRL values and to conduct a health risk assessment based on a hypothetical chemical accident scenario using both HGV types. By comparing and analyzing the differences in application between these two approaches, we aimed to evaluate the efficacy of conventional assessment methods and propose a more valid risk assessment strategy for short-term exposures to hazardous chemicals resulting from chemical accidents. To the best of our knowledge, this study is the first to empirically apply and compare RfC/RfD- and MRL-based health risk assessments in the context of a short-term chemical accident scenario.

## 2. Materials and Methods

### 2.1. Chemicals

To select target substances for the health risk assessment, we reviewed the 97 substances designated as accident preparedness chemicals under the Chemical Substances Control Act managed by the Ministry of Environment of Korea [9]. Accident preparedness chemicals are substances with high acute toxicity, explosiveness, or other hazardous properties, which make them more likely to cause chemical accidents or lead to large scale damage in the event of an incident. Therefore, these substances require special preparedness and management. From these, we identified substances for which both RfC/RfD and MRL values were available. We selected one substance with low persistence and another with high persistence to represent chemicals with varying environmental persistence. Consequently, toluene and formaldehyde were selected as the target substances for assessment. Toluene is characterized by high volatility and a short residence time in the environment [10], whereas formaldehyde can persist for a relatively long time in the atmosphere, with a half-life of up to 114 d, as photochemical reactions are limited under certain environmental conditions, delaying its degradation [11]. Additionally, these two substances have a high frequency of occurrence in actual chemical accidents. According to chemical accident statistics in the Korea from 2014 to July 2025, formaldehyde ranked 8th and toluene 16th [12]. These rankings underscore their representativeness as hazardous chemicals involved in chemical accidents.

### 2.2. Health Guidance Values

We reviewed the respective HGVs and the foundational studies for the selected substances (toluene and formaldehyde) used to derive these values. RfC and RfD values were sourced from the Integrated Risk Information System (IRIS) of the US EPA, and the corresponding MRLs were obtained from the ATSDR. Some acute, intermediate, or chronic MRL values were not available, as the ATSDR has not established them for the respective exposure durations. We also examined the exposure durations, test species, and experimental conditions used in the foundational studies for each HGV in order to compare their differences and evaluate their suitability for short-term health risk assessments.

### 2.3. Multimedia Environmental Dynamics Model

The multimedia environmental dynamics model developed by Lee et al. [13] is a FORTRAN-based model designed to simulate the environmental behavior of chemicals across air, soil, and water media following a chemical accident [4,14,15]. Operating under non-steady-state conditions, the model incorporates meteorological data (e.g., wind direction, wind speed, and precipitation) sourced from the Korea Meteorological Administration for the duration of the simulated accident. The modeled domain was set to a 15 km × 15 km area centered on the accident site, with a nested grid resolution of 100 m × 100 m.

The virtual accident scenario was based on the region with the highest frequency of chemical release incidents in Korea between January 2016 and April 2025, which, in this case, was Gyeonggi Province [12]. Among the 123 documented chemical accidents in this period, the scenario was modeled after the incident with the largest recorded release quantity (11 tons).

The endpoint of chemical dispersion was defined as the point at which the concentration of the released substance decreased to below its background level, i.e., 8.36 µg/m^3^ for toluene [16] and 4.80 µg/m^3^ for formaldehyde [17].

The final accident scenario was established as follows. At 12:00 p.m. on 3 July 2019, an accident occurred at a chemical plant in Ansan, Gyeonggi Province, resulting in the complete release of 11 tons of a chemical substance over the course of 1 h. It was assumed that the exposed population remained in the affected area throughout the entire persistence period of the chemical.

### 2.4. Non-Carcinogenic Health Risk Assessment

Based on the persistence durations estimated using the multimedia environmental dynamics model, toluene remained in the environment for 9 d, and formaldehyde persisted for 69 d. Accordingly, the acute MRL was applied to toluene, and the intermediate MRL was applied to formaldehyde (Table 1). The concept of the toxicity reference value (TRV) was introduced to facilitate a comprehensive comparison and evaluation of different HGVs. TRVs are categorized into inhalation exposure (TRV_inhal_) and oral exposure (TRV_oral_), defined as follows: TRV_inhal_ refers to a toxicity benchmark for inhalation exposure and is derived by converting RfC and MRL_inhal_ values using Equation (1); TRV_oral_ refers to the toxicity benchmark for oral exposure and is defined as either RfD or MRL_oral_.

(1) $${TRV}_{inhal\;}=\frac{RfC\;or\;{MRL}_{inhal}\times 20\;m^{3}/day\;}{70\;kg\;}$$

Here, *TRV_inhal_* is the toxicity reference value for inhalation exposure (mg/kg/d), *RfC* is the reference concentration (mg/m^3^), and *MRL_inhal_* is the minimal risk level for inhalation exposure (mg/m^3^).

**Table 1:** Health guidance values (HGVs) of target chemicals analyzed in this study.

Substance	Exposure Pathway	HGV	Duration	Value	Reference
Toluene	Inhalation	RfC ^a^	Chronic (lifespan)	5.00 mg/m^3^	[18]
		MRL ^b^	Acute (1–14 d)	7.54 mg/m^3^	[10]
	Oral	RfD ^c^	Chronic (lifespan)	0.08 mg/kg/d	[18]
		MRL	Acute (1–14 d)	0.8 mg/kg/d	[10]
Formaldehyde	Inhalation	RfC	Chronic (lifespan)	0.007 mg/m^3^	[19]
		MRL	Intermediate (15–364 d)	0.037 mg/m^3^	[20]
	Oral	RfD	Chronic (lifespan)	0.2 mg/kg/d	[21]
		MRL	Intermediate (15–364 d)	0.3 mg/kg/d	[20]

^a^ Reference concentration. ^b^ Minimal risk level. ^c^ Reference dose.

The assessment was stratified by the age groups 0–9 years, 10–18 years, 19–64 years, and ≥65 years. It was assumed that individuals engaged in typical daily activities and that exposure routes were evaluated through inhalation and incidental soil ingestion. This is due to chemicals released during accidents persisting in air or soil and being re-emitted, resulting in prolonged environmental contamination and ongoing human exposure [4,22]. Exposure factors used in the assessment are summarized in Table 2.

Inhalation exposure to the target chemicals was assessed for both outdoor and indoor environments. Outdoor exposure levels were determined based on the results of the multimedia environmental dynamics model. It was assumed that the released chemical substances infiltrated indoor environments for indoor exposure. Indoor concentrations were estimated using the indoor concentration prediction model developed by Park et al. [26] through meta-analysis, as expressed in Equation (2):

(2) $$C_{n\;indoor\;}=0.33C_{n\;outdoor}$$

where *C_n indoor_* is the concentration of indoor target chemical *n* days after accident occurrence (mg/m^3^) and *C_n outdoor_* is the concentration of outdoor target chemical *n* days after accident occurrence (mg/m^3^).

The total inhalation exposure dose (incorporating both indoor and outdoor concentrations) was calculated as the average daily dose (ADD; mg/kg/d) using Equation (3), under the assumption of 100% absorption of the inhaled chemical:

(3) $$ADD=\;\frac{\sum_{n=1}^{CLT}\left\{\left(C_{n\;outdoor\;}\times \;IHR\;\times \;OET\right)+\left(C_{n\;indoor\;}\times \;IHR\;\times \;IET\right)\right\}}{BW\;\times \;AT}$$

where *CLT* is the period until extinction of the target chemical in the environment (d), *IHR* is the inhalation rate (m^3^/d), *OET* is the outdoor exposure time (d), *IET* is the indoor exposure time (d), *BW* is body weight (kg), and *AT* is the average exposure time (d)

Oral exposure via soil ingestion was assessed by calculating the ADD using Equation (4), which also assumed a 100% absorption of the ingested chemical:

(4) $$ADD=\frac{\sum_{n=1}^{CLT}(C_{n\;soil}\;\times \;{ITR}_{soil}\;\times \;1\;day)}{BW\;\times \;AT}$$

where *C_n soil_* is the concentration of the target chemical in soil *n* days after accident occurrence (mg/kg) and *ITR_soil_* is the soil intake rate (kg/d).

The non-carcinogenic risk was assessed by calculating the hazard quotient (HQ) for each exposure route, as defined in Equation (5):

(5) $$HQ=\;\frac{ADD}{TRV}$$

by dividing the route-specific ADD by the corresponding TRV; an HQ > 1 indicates potential for adverse health effects associated with that particular exposure route [27].

## 3. Results and Discussion

### 3.1. Health Guidance Values

A review of the foundational studies used to derive inhalation-based RfC and MRL values for toluene revealed the following findings (Table 3). Both the RfC and chronic MRL were derived from occupational studies involving long-term repeated exposures for toluene, and the acute MRL was based on experimental results from a single acute exposure study. When comparing chronic values, the RfC for toluene was approximately 1.3 times higher than the chronic MRL.

The RfC for formaldehyde was derived from three short-term human exposure studies (≤2 weeks). On the other hand, acute, intermediate, and chronic MRLs were based on exposure durations of 2 h, 26 weeks, and 10.4 years, respectively (Table 4). Notably, the RfC for formaldehyde was approximately 0.7 times lower than its chronic MRL, indicating a more conservative RfC.

**Table 3:** Summary of key studies used to derive inhalation reference concentration (RfC) and minimal risk level (MRL) values for toluene.

Category	RfC	Acute MRL	Chronic MRL
Critical study	Multiple human studies (n = 10)	Little et al. [28]	Multiple human studies (n = 6)
Test subjects	Workers	Sensitive group	Workers
Exposure duration	≥1 year	20 min	13.5 years
Critical effect	Neurological effects	Neurological effects	Neurological effects
Point of departure	NOAEL (ADJ) ^a^, 12.21 ppm	LOAEL ^b^ 15 ppm	NOAEL (ADJ) ^c^, 10 ppm
Uncertainty factors	10 (intraspecies variation)	3 (LOAEL to NOAEL) 3 (intraspecies variation)	10 (intraspecies variation)
Value	5.00 mg/m^3^	7.54 mg/m^3^	3.77 mg/m^3^
Reference	[18]	[10]	[10]

^a^ Arithmetic mean of NOAELs (No-observed-adverse-effect levels) from 10 studies was adjusted from occupational exposure scenario to continuous exposure scenario. ^b^ Lowest-observed-adverse-effect level. ^c^ NOAEL was adjusted from occupational exposure scenario to continuous exposure scenario.

**Table 4:** Summary of key studies used to derive inhalation reference concentration (RfC) and minimal risk level (MRL) values for formaldehyde.

Category	RfC	Acute MRL	Intermediate MRL	Chronic MRL
Critical study	Krzyzanowski et al. [29]; Venn et al. [30]; Annesi-Maesano et al. [31]	Pazdrak et al. [32]	Rusch et al. [33]	Holmström et al. [34]
Test subjects	Children	Human	Cynomolgus monkey	Workers
Exposure duration	≤2 weeks	2 h	26 weeks	10.4 years
Critical effect	Pulmonary function decreases; Asthma prevalence or control; Allergic conditions	Nasal and eye irritation	Nasopharyngeal irritation; Nasal epithelial lesions	Nasal epithelium damage; Eyes and upper respiratory tract irritation
Point of departure	osRfC ^a^, 0.006–0.008 mg/m^3^ (midpoint = 0.007 mg/m^3^)	LOAEL ^b^, 0.4 ppm	NOAEL ^c^, 0.98 ppm	LOAEL, 0.24 ppm
Uncertainty factors	3 or 10 (intraspecies variation)	3 (LOAEL to NOAEL) 3 (intraspecies variation)	3 (interspecies variation) 10 (intraspecies variation)	3 (LOAEL to NOAEL) 10 (intraspecies variation)
Value	0.007 mg/m^3^	0.049 mg/m^3^	0.037 mg/m^3^	0.010 mg/m^3^
Reference	[19]	[20]	[20]	[20]

^a^ RfC derived from evidence of effects on specific organ or physiological system. ^b^ Lowest-observed-adverse-effect level. ^c^ No-observed-adverse-effect level.

For oral exposure, the RfD for toluene was derived from a 13-week sub-chronic repeated dose study due to the absence of adequate chronic data. An uncertainty factor (UF) of 10 was applied to extrapolate the findings to a chronic exposure context (Table 5). Acute and intermediate MRLs for toluene were based on exposure durations of 45 min and 28 d, respectively.

In the case of formaldehyde, both the RfD and the chronic MRL were derived from the same chronic data source and were assigned identical values, and the intermediate MRL was derived from a 4-week study (Table 6).

The analysis of the exposure durations used in the foundational studies for each HGV revealed that, except for the RfC for formaldehyde and the RfD for toluene, all HGVs were derived under research conditions comparable to their respective exposure durations. This alignment served to minimize uncertainty. The rationale for this may be that certain toxic effects may manifest during low-dose chronic exposure but not during high-dose acute exposure [42]. Therefore, selecting reference studies with comparable exposure durations is essential to avoid underestimating or overestimating risk. The toxicity from single exposures differs significantly from that of repeated exposures for several chemicals. A single exposure can produce severe effects in some cases, whereas the same cumulative dose distributed over time may produce no effects at all [43]. In line with this, the RfC and chronic MRL for toluene and the RfD for formaldehyde were based on studies involving >1 year of chronic exposure. The RfD for toluene was derived from a 13-week sub-chronic study, with a UF of 10 applied in order to extrapolate chronic exposure conditions. Notably, the RfC for formaldehyde, although classified as a chronic reference value, was based on three human studies with exposure durations of <2 weeks. As clarified by the US EPA [19], acute data may be prioritized over chronic data when exposure elicits stronger effects or when sensitive populations are disproportionately affected. With this exception, RfC and RfD values are, in principle, based on chronic exposure data. Given the differing toxicity mechanisms between acute and chronic exposures, applying RfC/RfD values to short-term exposure scenarios may be inappropriate. In contrast, MRLs are clearly categorized by exposure duration (i.e., acute [1–14 d], intermediate [15–364 d], and chronic [≥1 year]), making them more appropriate for short-term scenarios such as chemical accidents. Acute MRLs were derived from acute exposure studies (e.g., 20 min, 2 h, and 45 min), and intermediate MRLs were based on studies of intermediate duration (e.g., 26 weeks, 28 d, and 4 weeks). Therefore, the use of acute and intermediate MRLs in such contexts is considered a more valid approach to risk assessment. Additionally, the similarity in magnitude between RfC and chronic MRL values for both substances (1.3- and 0.7-fold variations for toluene and formaldehyde, respectively) suggested consistency between the two HGVs. In particular, the fact that the RfD and chronic MRL for formaldehyde were identical (being derived from the same dataset) supported the methodological validity and scientific credibility of MRL derivation when sufficient toxicological data were available.

### 3.2. Non-Carcinogenic Health Risk Assessment

To examine the differences in HQs resulting from the application of each HGV, we derived age-specific HQs for inhalation and ingestion exposures based on both RfC/RfD and MRL values (Table 7). The comparative analysis of RfC/RfD- and MRL-based HQs enabled an age-stratified assessment of potential health risks. For inhalation exposure, both RfC- and MRL-based HQs for toluene and formaldehyde were > 1 at their respective maximums, indicating a potential health concern. In terms of oral exposure, although RfD-based HQs were higher than MRL-based HQs for both chemicals, all values remained <1 across all age groups, suggesting no significant health risk via ingestion for either substance. These results confirm that RfC/RfD-based HQs were consistently higher than MRL-based HQs for all age groups and both substances. This implied that RfC/RfD values tended to yield more conservative risk estimates. Therefore, sole reliance on RfC/RfD, as is common in several studies, may result in overly conservative assessments and could lead to unnecessary or excessive regulatory actions in situations where the actual health risk is low.

**Figure 1:**
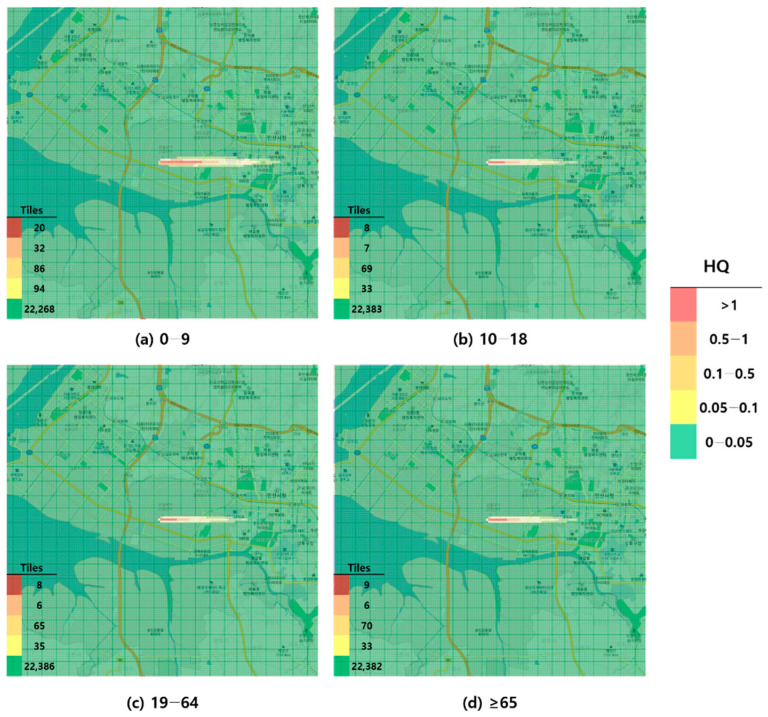
Hazard map of toluene inhalation exposure based on reference concentration (RfC)-derived hazard quotient (HQ) values by age group; (**a**) 0–9 years; (**b**) 10–18 years; (**c**) 19–64 years; and (**d**) ≥65 years.

**Figure 2:**
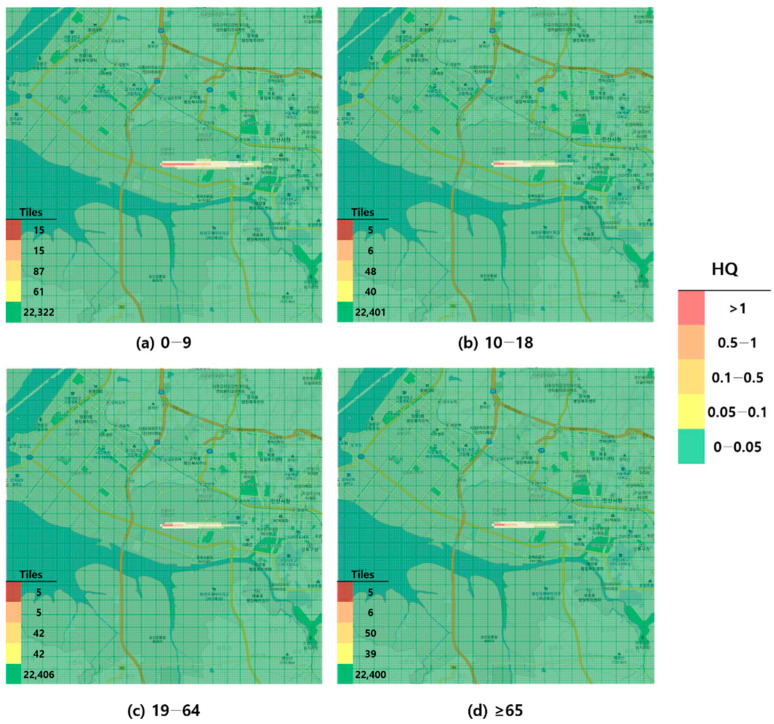
Hazard map of toluene inhalation exposure based on acute minimal risk level (MRL)-derived hazard quotient (HQ) values by age group; (**a**) 0–9 years; (**b**) 10–18 years; (**c**) 19–64 years; and (**d**) ≥65 years.

**Figure 3:**
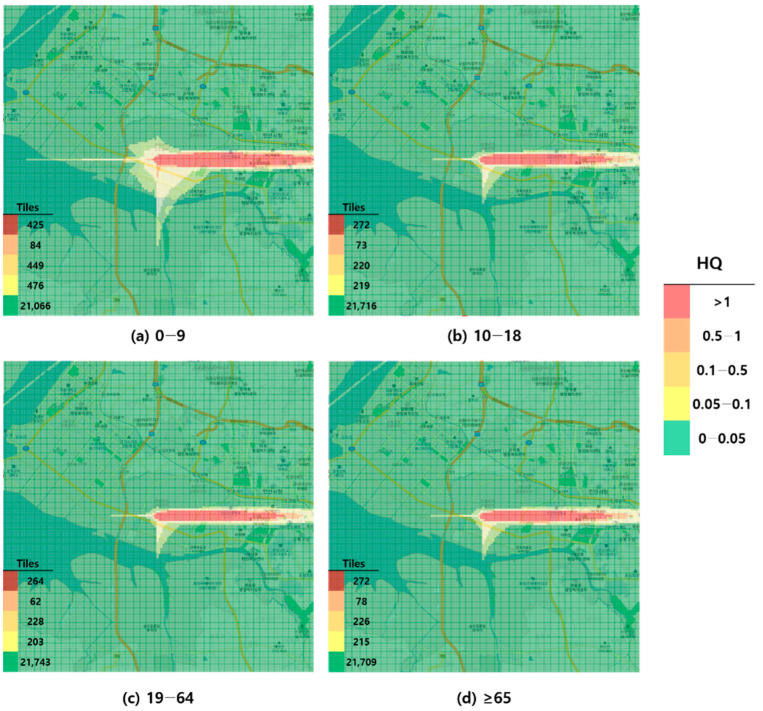
Hazard map of formaldehyde inhalation exposure based on reference concentration (RFC)-derived hazard quotient (HQ) values by age group; (**a**) 0–9 years; (**b**) 10–18 years; (**c**) 19–64 years; and (**d**) ≥65 years.

**Figure 4:**
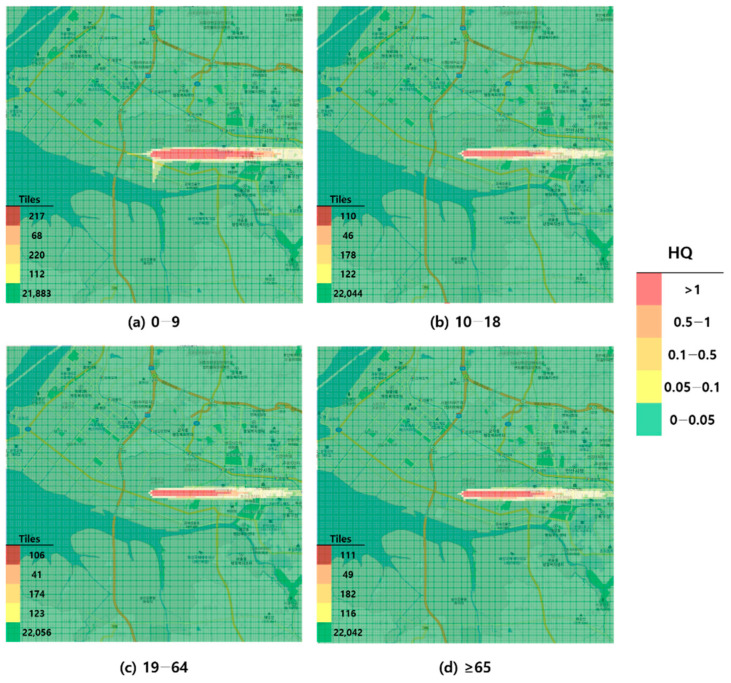
Hazard map of formaldehyde inhalation exposure based on intermediate minimal risk level (MRL)-derived hazard quotient (HQ) values by age group; (**a**) 0–9 years; (**b**) 10–18 years; (**c**) 19–64 years; and (**d**) ≥65 years.

When visualized through hazard maps illustrating the spatial distribution of HQ values for inhalation exposure, it was evident that RfC-based assessments identified more areas of potential concern (HQ > 1) than MRL-based assessments (Figure 1, Figure 2, Figure 3 and Figure 4). The greatest discrepancy in the number of tiles (grid size: 100 m × 100 m) exceeding an HQ of 1 was observed in the 0–9 years age group. Due to the higher HQ values calculated using RfCs, an additional five tiles (0.05 km^2^) for toluene and 208 tiles (2.08 km^2^) for formaldehyde were classified as areas of potential concern relative to the MRL-based evaluation. The 2.08 km^2^ of additional area corresponds to approximately 41% of the average administrative district area in Korea (5.10 km^2^), where the average population per district is 19,716 [44]. These findings highlighted that the selection of HGVs can significantly influence the extent of areas of potential concern under identical exposure conditions, potentially altering health risk determinations for large population groups. This underscores the critical importance of appropriate HGV selection in risk assessment frameworks.

**Table 7:** Hazard quotient (HQ) results for each exposure route by age group.

Substance	TRV ^a^	Age	Inhalation			Ingestion		
			Min	Max	Median	Min	Max	Median
Toluene	RfC	0–9	8.86 × 10^−12^	1.37 × 10^1^	1.25 × 10^−7^	5.47 × 10^−19^	8.46 × 10^−7^	7.73 × 10^−15^
		10–18	2.91 × 10^−12^	4.49 × 10^0^	4.11 × 10^−8^	3.79 × 10^−20^	5.86 × 10^−8^	5.35 × 10^−16^
		19–64	2.68 × 10^−12^	4.13 × 10^0^	3.78 × 10^−8^	3.16 × 10^−20^	4.89 × 10^−8^	4.47 × 10^−16^
		≥65	3.00 × 10^−12^	4.62 × 10^0^	4.23 × 10^−8^	3.51 × 10^−20^	5.43 × 10^−8^	4.96 × 10^−16^
	Acute MRL	0–9	5.88 × 10^−12^	9.07 × 10^0^	8.30 × 10^−8^	5.47 × 10^−20^	8.46 × 10^−8^	7.73 × 10^−16^
		10–18	1.93 × 10^−12^	2.98 × 10^0^	2.73 × 10^−8^	3.79 × 10^−21^	5.86 × 10^−9^	5.35 × 10^−17^
		19–64	1.77 × 10^−12^	2.74 × 10^0^	2.51 × 10^−8^	3.16 × 10^−21^	4.89 × 10^−9^	4.47 × 10^−17^
		≥65	1.99 × 10^−12^	3.07 × 10^0^	2.81 × 10^−8^	3.51 × 10^−21^	5.43 × 10^−9^	4.96 × 10^−17^
Formaldehyde	RfC	0–9	1.74 × 10^−5^	1.28 × 10^3^	3.90 × 10^−3^	6.02 × 10^−16^	4.41 × 10^−8^	1.35 × 10^−13^
		10–18	5.72 × 10^−6^	4.19 × 10^2^	1.28 × 10^−3^	4.17 × 10^−17^	3.05 × 10^−9^	9.35 × 10^−15^
		19–64	5.26 × 10^−6^	3.85 × 10^2^	1.18 × 10^−3^	3.48 × 10^−17^	2.55 × 10^−9^	7.81 × 10^−15^
		≥65	5.89 × 10^−6^	4.31 × 10^2^	1.32 × 10^−3^	3.86 × 10^−17^	2.83 × 10^−9^	8.66 × 10^−15^
	Intermediate MRL	0–9	2.48 × 10^−6^	2.43 × 10^2^	7.42 × 10^−4^	4.01 × 10^−16^	2.94 × 10^−8^	9.00 × 10^−14^
		10–18	8.16 × 10^−7^	7.96 × 10^1^	2.43 × 10^−4^	2.78 × 10^−17^	2.04 × 10^−9^	6.23 × 10^−15^
		19–64	7.50 × 10^−7^	7.32 × 10^1^	2.24 × 10^−4^	2.32 × 10^−17^	1.70 × 10^−9^	5.21 × 10^−15^
		≥65	1.12 × 10^−6^	8.20 × 10^1^	2.51 × 10^−4^	2.57 × 10^−17^	1.89 × 10^−9^	5.78 × 10^−15^

^a^ Toxicity reference value.

Accordingly, this study provides empirical evidence that MRLs reflecting exposure duration are more appropriate than the chronically oriented RfC/RfD values in short-term chemical accident scenarios. To the best of our knowledge, this is the first study to apply MRLs in short-term chemical accident scenarios and directly compare them with RfC/RfD values. It emphasizes the influence of HGV selection on risk characterization and provides a scientific foundation for improving short-term exposure assessment frameworks. These findings are expected to support the estimation of damage following chemical accidents and the development of appropriate compensation and response measures.

However, this study has two limitations. First, the results were derived from an assessment of only two substances: toluene and formaldehyde. In reality, a wide range of hazardous chemicals can be released during chemical accidents, and it is difficult to generalize conclusions drawn from a limited number of substances to all chemical types. Therefore, further evaluations involving chemicals with diverse toxicological mechanisms and physicochemical properties are necessary to generalize and refine the framework for health risk assessment under short-term exposure conditions. Second, the analysis was based on a single hypothetical accident scenario. As such, the study may not fully capture the variability in chemical dispersion patterns and risk assessment outcomes under different geographical, meteorological, and release conditions. Thus, further research incorporating a variety of scenarios is needed to enhance the applicability of the findings.

## 4. Conclusions

To date, health risk assessments for hazardous chemical exposures resulting from chemical accidents have commonly relied on chronic-based benchmarks such as the RfC and RfD, even in short-term exposure scenarios. However, this approach has inherent limitations, as it does not adequately reflect the unique characteristics of short-term exposure. To address these limitations, this study aimed to evaluate the efficacy of existing assessment methods and propose a more suitable risk assessment approach for short-term exposure to hazardous chemicals.

Examination of exposure durations in the foundational studies revealed that most HGVs were based on studies closely aligned with their designated exposure durations. RfC/RfD values are fundamentally derived from chronic data. Therefore, they have inherent limitations when applied directly to short-term exposure scenarios, as they may not sufficiently reflect the toxicological mechanism involved in short-term exposures. In contrast, MRLs are stratified by exposure duration, making them more suitable benchmarks for short-term risk assessments.

As a result of comparing the HQs, the MRL-based HQs were lower than the RfC/RfD-based HQs across all exposure routes (inhalation and oral) for both toluene and formaldehyde. This suggests that the RfC/RfD-based HQs provide a relatively conservative assessment. In particular, for formaldehyde, the use of RfC instead of MRL resulted in the identification of an additional 2.08 km^2^ of land (equivalent to 41% of the average area of an administrative district in Korea) as a potential concern (HQ > 1). This discrepancy underscores how the selection of HGVs can significantly affect risk determinations and potentially impact numerous residents.

These findings provide empirical evidence that the application of MRLs is more appropriate than RfC/RfD values in short-term health risk assessments and establish a scientific foundation for improving short-term risk assessment frameworks and developing rational risk management strategies. To the best of our knowledge, this study is the first to empirically apply and compare MRL- and RfC/RfD-based health risk assessments in the context of a short-term chemical accident scenario. In addition, the findings of this study are expected to contribute to estimating the scale of damage following chemical accidents as well as to the development of compensation and response measures. However, as this study was based on a limited number of substances and a single hypothetical accident scenario, further research that incorporates a wider range of chemicals and exposure conditions is necessary.

## Figures and Tables

**Table 2:** Exposure factors used in this study.

Category	Age Group (Years)	Value	Reference
Body weight (kg)	0–9	13.21	[23]
	10–18	54.53	[23]
	19–64	65.30	[24]
	≥65	58.85	[24]
Inhalation rate (m^3^/d)	0–9	10.27	[23]
	10–18	14.03	[23]
	19–64	14.61	[24]
	≥65	14.60	[24]
Soil intake rate (mg/d)	0–9	35	[23]
	10–18	10	[25]
	19–64	10	[25]
	≥65	10	[25]
Outdoor exposure time (d)	0–9	0.063	[23]
	10–18	0.058	[23]
	19–64	0.090	[24]
	≥65	0.095	[24]
Indoor exposure time (d)	0–9	0.94	[23]
	10–18	0.94	[23]
	19–64	0.91	[24]
	≥65	0.90	[24]
Average exposure time (d)	Toluene	9	This study
	Formaldehyde	69	This study

**Table 5:** Summary of key studies used to derive oral reference dose (RfD) and minimal risk level (MRL) values for toluene.

Category	RfD	Acute MRL	Intermediate MRL
Critical study	National Toxicology Program [35]	Dyer et al. [36]	Hsieh et al. [37,38,39]
Test subjects	Rats and mice	Rats	Mouse
Exposure duration	13 weeks	45 min	28 d
Critical effect	Increased kidney weight	Neurological effects	Immune depression
Point of departure	BMDL ^a^, 238 mg/kg/d	LOAEL ^b^, 250 mg/kg	NOAEL ^c^, 22 mg/kg/d
Uncertainty factors	10 (interspecies variation) 10 (intraspecies variation) 10 (sub-chronic to chronic) 3 (database insufficiencies)	3 (LOAEL to NOAEL) 10 (interspecies variation) 10 (intraspecies variation)	10 (interspecies variation) 10 (intraspecies variation)
Value	0.08 mg/kg/d	0.8 mg/kg/d	0.2 mg/kg/d
Reference	[18]	[10]	[10]

^a^ Lower one-sided confidence limit on benchmark dose. ^b^ Lowest-observed-adverse-effect level. ^c^ No-observed-adverse-effect level.

**Table 6:** Summary of key studies used to derive oral reference dose (RfD) and minimal risk level (MRL) values for formaldehyde.

Category	RfD	Intermediate MRL	Chronic MRL
Critical study	Til et al. [40]	Til et al. [41]	Til et al. [40]
Test subjects	Rats	Rats	Rats
Exposure duration	24 months	4 weeks	24 months
Critical effect	Reduced weight gain; Histopathology in rats	Gastrointestinal effects	Gastrointestinal effects
Point of departure	NOAEL ^a^, 15 mg/kg/d	NOAEL, 25 mg/kg/d	NOAEL, 15 mg/kg/d
Uncertainty factors	10 (interspecies variation) 10 (intraspecies variation)	10 (interspecies variation) 10 (intraspecies variation)	10 (interspecies variation) 10 (intraspecies variation)
Value	0.2 mg/kg/d	0.3 mg/kg/d	0.2 mg/kg/d
Reference	[21]	[20]	[20]

^a^ No-observed-adverse-effect level.

## Data Availability

Data are contained within the article.

## Abbreviations

The following abbreviations are used in this manuscript:

ADD	Average daily dose
ARE	Acute reference exposure
AT	Average exposure time
ATSDR	Agency for Toxic Substances and Disease Registry
BMDL	A lower one-sided confidence limit on the benchmark dose
BW	Body weight
C	Concentration
CLT	Period until extinction of the target chemical in the environment
HGV	Health guidance value
HQ	Hazard quotient
IET	Indoor exposure time
IHR	Inhalation rate
IRIS	Integrated Risk Information System
ITR	Intake rate
LOAEL	Lowest-observed-adverse-effect level
MoE	Ministry of the Environment
MOIS	Ministry of Interior and Safety
MRL	Minimal risk level
NICS	National Institute of Chemical Safety
NIER	National Institute of Environmental Research
NOAEL	No-observed-adverse-effect level
OET	outdoor exposure time
RfC	Reference concentration
RfD	Reference dose
TRV	Toxicity reference value
UF	Uncertainty factor
US EPA	United States Environmental Protection Agency
